# The *Arabidopsis* O-fucosyltransferase SPINDLY regulates root hair patterning independently of gibberellin signaling

**DOI:** 10.1242/dev.192039

**Published:** 2020-10-09

**Authors:** Krishna Vasant Mutanwad, Isabella Zangl, Doris Lucyshyn

**Affiliations:** Institute of Molecular Plant Biology, Department of Applied Genetics and Cell Biology, University of Natural Resources and Life Sciences, Muthgasse 18, 1190 Vienna, Austria

**Keywords:** *Arabidopsis*, Root hair patterning, Glycosylation, Gibberellin

## Abstract

Root hairs are able to sense soil composition and play an important role in water and nutrient uptake. In *Arabidopsis thaliana*, root hairs are distributed in the epidermis in a specific pattern, regularly alternating with non-root hair cells in continuous cell files. This patterning is regulated by internal factors such as a number of hormones, as well as by external factors like nutrient availability. Thus, root hair patterning is an excellent model for studying the plasticity of cell fate determination in response to environmental changes. Here, we report that loss-of-function mutants for the Protein O-fucosyltransferase SPINDLY (SPY) show defects in root hair patterning. Using transcriptional reporters, we show that patterning in *spy-22* is affected upstream of GLABRA2 (GL2) and WEREWOLF (WER). O-fucosylation of nuclear and cytosolic proteins is an important post-translational modification that is still not very well understood. So far, SPY is best characterized for its role in gibberellin signaling via fucosylation of the growth-repressing DELLA protein REPRESSOR OF *ga1-3* (RGA). Our data suggest that the epidermal patterning defects in *spy-22* are independent of RGA and gibberellin signaling.

## Abstract (precis)

**Summary:** The O-fucosyltransferase SPY regulates root hair patterning independently of DELLA proteins and gibberellin signaling when modulating epidermal tissue patterning in *Arabidopsis* *thaliana* roots.

## INTRODUCTION

Post-translational modifications (PTMs) dynamically modulate various physiological and morphological events throughout the lifespan of plants (Millar et al., 2019). O-glycosylation of nuclear and cytosolic proteins is one such PTM, and plants carry two O-glycosyltransferases responsible for these modifications: the protein O-fucosyltransferase (POFUT) SPINDLY (SPY); and the O-GlcNAc transferase (OGT) SECRET AGENT (SEC) (Hartweck et al., 2002; Olszewski et al., 2010; Zentella et al., 2016, 2017). These proteins regulate significant events in plants, from embryo development to the determination of flowering time and flower development (Hartweck et al., 2002, 2006). *spy* mutants were initially identified due to their resistance to the gibberellin (GA) biosynthesis inhibitor paclobutrazol, leading to constitutively active GA signaling (Jacobsen and Olszewski, 1993; Swain and Olszewski, 1996). Further studies reported that SPY and SEC are involved in GA signaling via modification of the growth-repressing DELLA protein RGA (REPRESSOR OF *ga1-3*) (Silverstone et al., 2007; Zentella et al., 2016, 2017). *spy* mutants display various phenotypic traits, such as early flowering, early phase transitions, partial male sterility, abnormal trichome formation and disordered phyllotaxy (Silverstone et al., 2007). Recently, SEC also was reported to be involved in delaying flowering time in *Arabidopsis thaliana* (Xing et al., 2018). The majority of the studies thus have focused on the role of O-glycosylation in aerial tissue development and the subsequent phenotypes are often attributed to its participation in GA signaling. SEC and SPY are also active in roots; however, their impact on root development and morphogenesis is largely unexplored (Hartweck et al., 2006; Silverstone et al., 2007; Swain et al., 2002).

Tissue morphology and cellular organization are decisive for root development in *Arabidopsis thaliana*. Epidermal tissue comprises hair-forming trichoblast cells and non-hair-forming atrichoblast cells (Dolan et al., 1993; Löfke et al., 2015; Scheres and Wolkenfelt, 1998). The arrangement of the hair and non-hair cells is established around the single ring-like layer of cortex cells. A hair cell arises at the junction between and is connected to two cortical cells, whereas a non-hair cell is usually adhered to only a single cortex cell. Moreover, hair cells are generally separated by non-hair cells between them (Balcerowicz et al., 2015; Dolan et al., 1994; Salazar-Henao et al., 2016). Various transcription factors such as GLABRA2 (GL2), WEREWOLF (WER) and CAPRICE (CPC) are responsible for determination of epidermal cell patterning in *Arabidopsis thaliana*. GL2 and WER regulate the establishment of non-hair cells (Lee and Schiefelbein, 1999; Masucci et al., 1996), whereas CPC activity is required for the formation of hair cells (Wada et al., 1997). GL2 expression is promoted by WER via the formation of a multiprotein complex comprising TRANSPARENT TESTA GLABRA (TTG1), GLABRA3 (GL3) and ENHANCER OF GLABRA3 (EGL3) (Bernhardt et al., 2003; Schiefelbein et al., 2014). Furthermore, GL2 establishes non-hair cell fate by suppressing the expression of root hair-promoting basic helix-loop-helix (bHLH) transcription factors such as ROOT HAIR DEFECTIVE 6 (RHD6), RHD6-LIKE1 (RSL1), RSL2, Lj-RHL1-LIKE1 (LRL1) and LRL2 (Balcerowicz et al., 2015; Masucci and Schiefelbein, 1996). On the contrary, in root hair cells, expression of WER is strongly reduced. This allows CPC or its paralogs ENHANCER OF TRY AND CPC 1 (ETC1), ETC3 or TRYPTICHON (TRY) to take its place in the TTG1/EGL3/GL3 complex, resulting in negative regulation of GL2 and de-repression of root hair-promoting genes, thus establishing root hair cell fate (Lee and Schiefelbein, 2002; Salazar-Henao et al., 2016).

Root hair development is dynamically controlled by environmental factors such as reactive oxygen species (ROS) and pH (Monshausen et al., 2007). Furthermore, availability of mineral nutrients such as inorganic phosphate (Pi) and iron (Fe) in the surroundings also modulates the development and morphology of root hairs (Janes et al., 2018; Müller and Schmidt, 2004; Salazar-Henao et al., 2016). Similarly, phytohormones such as auxin, ethylene and brassinosteroids are known to influence root hair patterning and development (Balcerowicz et al., 2015; Borassi et al., 2020; Kuppusamy et al., 2009; Liu et al., 2018; Shibata and Sugimoto, 2019). However, a role for gibberellin (GA) in epidermis morphology, root hair formation and development has not been described as yet, nor a potential role of the O-glycosyltransferases SPY and SEC in this context. *spy* mutants have been previously reported to display an extra layer of cortex cells, the middle cortex (MC), a phenotype associated with high level ROS signaling (Cui and Benfey, 2009; Cui et al., 2014). Beyond this, root tissue morphology of *spy* and *sec* mutants is largely unexplored. Hence, we initiated the investigation of the role of SPY and SEC in root development and tissue patterning, also in relation to GA signaling. Here, we show that epidermis morphology and root hair patterning is altered in *spy*, but not in *sec*, mutants. Using a set of reporter constructs, we established that SPY regulates patterning upstream of WER. However, we did not find any evidence for an involvement of GA signaling, indicating that SPY regulates root hair patterning independently of DELLA proteins and GA signaling.

## RESULTS

### The *Arabidopsis thaliana* protein O-fucosyltransferase mutant *spy-22* has larger root apical meristems

**Fig. 1.**
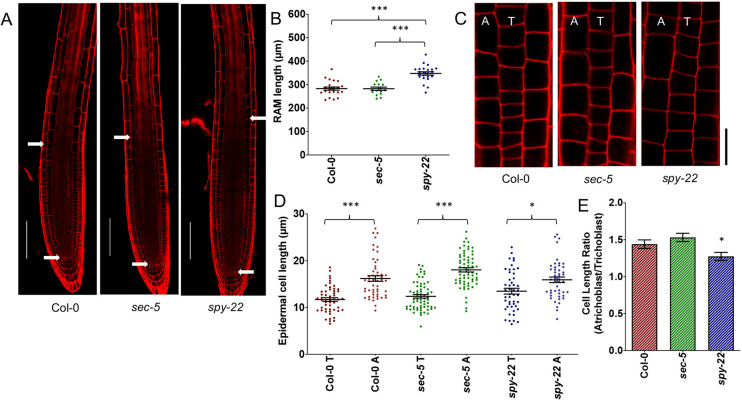
***spy-22* mutants display longer root apical meristems and reduced difference between atrichoblast and trichoblast cells.** (A) Longitudinal cross-section images of 7-day-old seedlings mounted in PI. Meristem size was defined as the distance from the quiescent center to first uppermost cortical cell, which was twice as long as wide, as indicated by white arrows. Scale bars: 100 μm. (B) *spy-22* roots display significantly longer meristems compared with Col-0 and *sec-5* (*n*=16-23). (C) The epidermal layer in the late meristematic region of 7-day-old seedlings mounted in PI. Lengths of four consecutive cells in neighboring (tricho/atrichoblast) files in the late meristem were measured. A, atrichoblasts; T, trichoblasts. Scale bar: 20 μm. (D) Atricho- and trichoblast cell length in Col-0, *sec-5* and *spy-22* (*n*=47-64). T, trichoblasts; A, atrichoblasts. (E) The ratio of the epidermal cell lengths of atrichoblasts/trichoblasts is lower in *spy-22* compared with *sec-5* and Col-0. For statistical analysis, one-way ANOVA with Tukey's multiple comparison was performed for B and D. Student's *t*-test was carried out for E (***P≤0.001, *P≤0.05). Data from three independent biological repeats are shown.

In order to investigate the involvement of O-glycosylation in *Arabidopsis thaliana* root development we analyzed various morphological phenotypes of the T-DNA insertion lines *spy-22* and *sec-5* in comparison with wild-type Col-0. SPY and SEC regulate GA signaling by modifying the DELLA protein RGA (Silverstone et al., 2007; Zentella et al., 2016, 2017) and *spy*-mutants display constitutive GA-signaling phenotypes (Jacobsen and Olszewski, 1993). GA-deficient mutants such as *ga1-3* are reported to have a reduced root apical meristem (RAM) size (Achard et al., 2009). To analyze whether O-glycosylation is involved in GA-dependent regulation of RAM size, we measured the RAM of 7-day-old seedlings – the region from quiescent center to the uppermost first cortical cell, which is twice as long as wide (Feraru et al., 2019). We observed that *spy-22* mutants displayed a significantly longer meristem (347.6±34.65 µm) compared with the wild-type Col-0 (283.6±31.92 µm) and *sec-5* (282.4±27.51 µm) (Fig. 1A,B). On counting the number of epidermal cells in the meristem, we found that the number of cells correlated with meristem size, showing a higher number of cells in *spy-22* (39.10±4.599) compared with Col-0 (29.05±3.965) and *sec-5* (28.92±5.008) (Fig. S1). This result is in line with the effect of increased GA signaling on cell division and meristem size (Achard et al., 2009).

In addition to cell number, the patterning and distribution of atrichoblasts (non-hair) and trichoblast (hair) cells of the epidermis is also crucial to determining the size of the meristematic region in *Arabidopsis thaliana* (Löfke et al., 2013). While analyzing our mutants, we observed that the difference between atricho- and trichoblast cell sizes was reduced in *spy-22* mutants compared with wild type and *sec-5*. To quantify that, we measured the lengths of the last four consecutive cells in adjacent (trichoblast and atrichoblast) cell files in the epidermis, marking the transition from the root meristem to the differentiation zone (Löfke et al., 2015). We noted that the atrichoblast cells in Col-0 and *sec-5* (16.21±4.30 µm and 18.05±3.62 µm, respectively) were significantly longer than trichoblast cells (11.70±2.81 µm and 12.38±2.95 µm, respectively). In *spy-22*, atrichoblast cells (15.92±4.08 µm) were only slightly longer than cells in corresponding trichoblast files (13.49±4.30 µm) (Fig. 1C,D). This difference was clearly reflected in a lower ratio of atrichoblast/trichoblast cell length in *spy-22* (1.27) compared with Col-0 (1.44) and *sec-5* (1.53) (Fig. 1E). Taken together, we observed both an increase in cell number, as well as an altered distribution of atrichoblast/trichoblast cell length in *spy-22*, resulting in an increase of root meristem size.

### *spy* mutants display ectopic root hairs

**Fig. 2.**
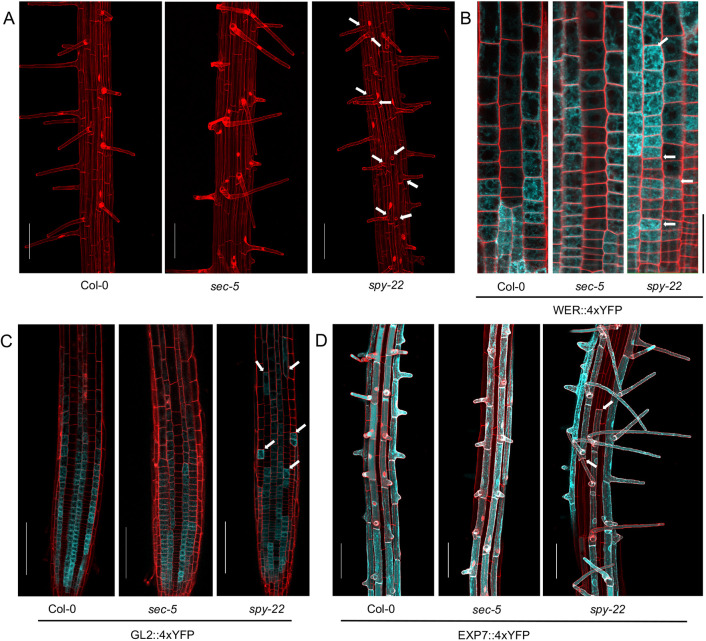
***spy-22* forms ectopic root hairs.** (A) Maximum projection of *z* stacks to visualize root hair patterning of 7-day-old seedlings. Scale bars: 100 μm. (B) WER::4xYFP expression in the epidermal cells in the meristem region. YFP signal in *spy-22* is unevenly distributed within the same cell file. Scale bar: 50 μm. (C) Expression of GL2::4xYFP visualized in atrichoblasts. Expression in *spy-22* indicates the presence of trichoblast cells in the atrichoblast cell file, and vice versa. Scale bars: 100 μm. (D) EXP7 is exclusively expressed in root hair cells. YFP signal indicates EXP7 promoter activity is not uniform within cell files in *spy-22*, suggesting the presence of non-hair cells in a hair cell file, and vice versa. Scale bars: 100 μm. Representative pictures of three biological repeats are shown. Arrows indicate irregular patterning within cell files.

**Fig. 3.**
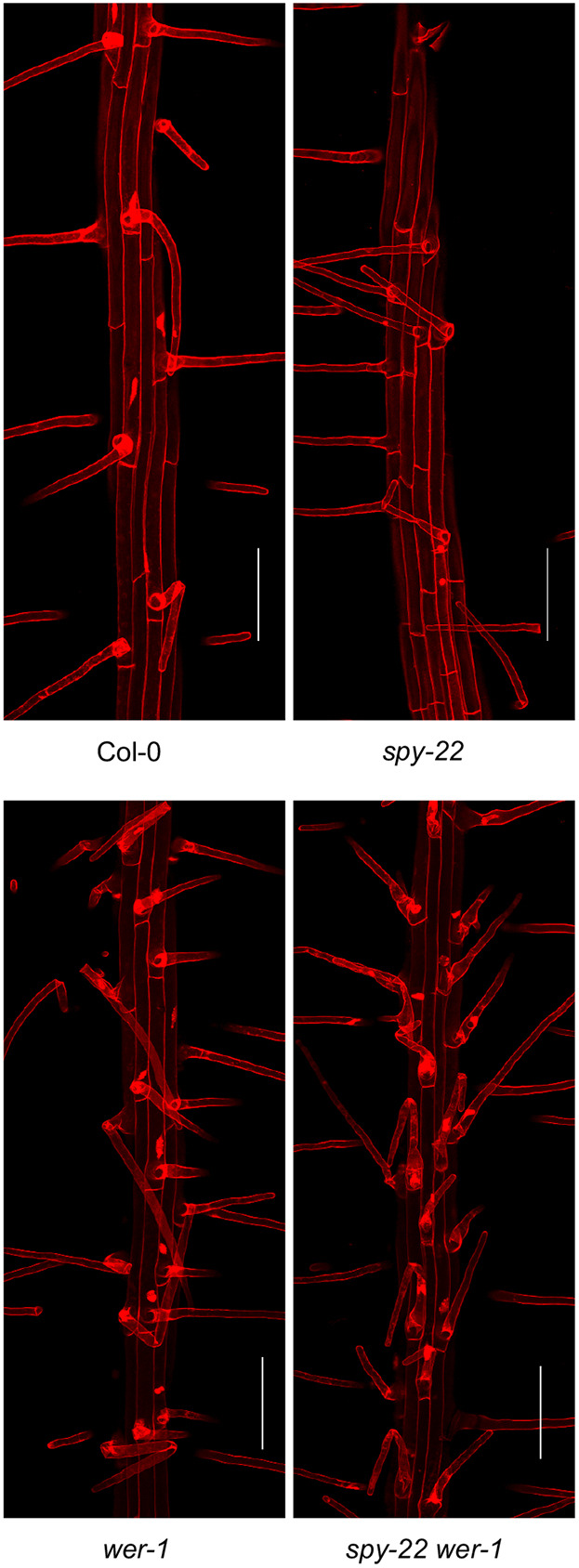
***wer-1* phenotype is epistatic in *spy-22 wer-1*.** Maximum projection of *z* stacks to visualize root hair patterning of Col-0, *wer-1*, *spy-22* and *spy-22 wer-1*. In loss-of-function *wer-1* mutants, all epidermal cells achieve hair cell identity, while root hair patterning in *spy-22* is patchy. *spy-22 wer-1* displays a phenotype similar to *wer-1*, where all epidermal cells take the hair cell identity. Scale bars: 100 µm.

The atypical atrichoblast to trichoblast morphology in *spy-22* led us to explore the consequences of this observation on root hair development in fully differentiated epidermis cells. In *spy-22*, we frequently observed the appearance of two trichoblast cells developing root hairs adjacent to each other, indicating ectopic root hair formation, while in Col-0 and *sec-5* root hair, cell files were always separated from each other by a non-hair cell file (Fig. 2A). The underlying cause for the appearance of ectopic root hairs in *spy-22* was further analyzed with the help of reporter lines. We used cell type-specific promoter-YFP fusions as described previously (Marquès-Bueno et al., 2016) to monitor the expression of transcription factors implicated in root hair patterning at different stages of development. We initially targeted WER, which is involved at an early stage of non-hair cell determination and is expressed strongly in atrichoblast cells and weakly in trichoblasts (Lee and Schiefelbein, 1999). On crossing the WER::4xYFP reporter with *spy-22* and *sec-5*, we observed an uneven signal distribution within single cell files in *spy-22* (Fig. 2B). We also crossed our lines to GL2::4xYFP, which in the wild type is exclusively expressed in the atrichoblasts in the cell division and transition zone. Although in Col-0 and *sec-5* a regular pattern of reporter gene expression was observed, GL2 expression in *spy-22* was very patchy, potentially underlying the formation of ectopic trichoblasts within non-hair cell files, and vice versa (Fig. 2C). We next employed a reporter that is active in differentiated root hair cells, to determine whether expression patterns in the meristematic and transition zone match the patterning of developed root hairs in the differentiation zone. EXP7 is expressed specifically in root hair cells. In EXP7::4xYFP *spy-22* we observed non-hair cells without signal within YFP-positive root hair cell files, and vice versa, an aberration in reporter expression that we did not detect in the Col-0 or *sec-5* background (Fig. 2D). Taken together, these results suggest that SPY regulates root hair patterning upstream of WER. Furthermore, a cross between *spy-22* and *wer-1* exclusively forms trichoblast cells, with every epidermal cell in *spy-22 wer-1* displaying root hairs in all cell files, as seen in *wer-1* (Fig. 3).

In wild-type conditions, it is known that hair cells develop at the junction of two cortex cells, whereas a non-hair cell is in contact with only a single underlying cortex cell (Dolan et al., 1994). In *spy-22*, we observed that root hair cells were frequently adhered to only a single underlying cortex cell (Fig. S2A). Additionally, it has previously been shown that *spy*-mutants generate an additional layer of root cortex cells, which is attributed to constitutively increased ROS signaling (Cui and Benfey, 2009; Cui et al., 2014). This middle cortex between the cortex and the endodermis was also clearly visible in *spy-22* (Fig. S2B). When crossing our lines with SCR::4xYFP to visualize specifically the endodermis, we could confirm the increase in middle cortex formation and clearly distinguish ectopic cell file formation from the endodermis, as seen before (Cui and Benfey, 2009), but there is no indication for a defect in endodermis formation in *spy-22* (Fig. S2C).

### Epidermal cell patterning and ectopic root hair formation in *spy-22* is independent of gibberellin signaling

**Fig. 4.**
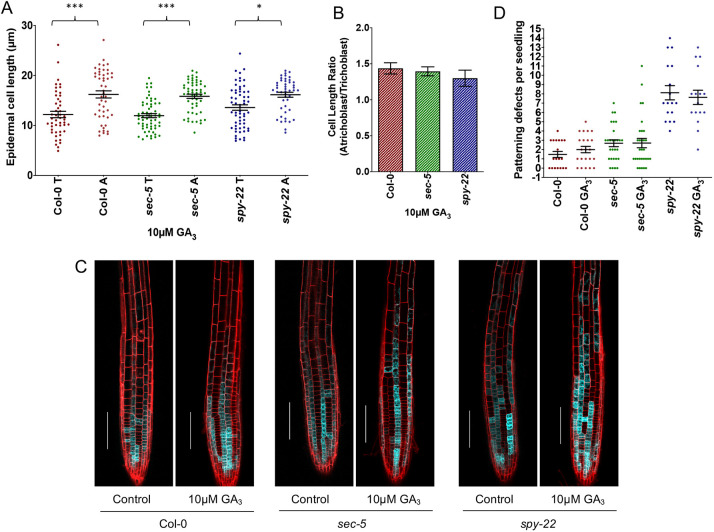
**Epidermal patterning in *spy-22* is independent of GA.** (A) Epidermal cell length of 7-day-old Col-0, *sec-5* and *spy-22* seedlings grown on ½ MS supplemented with 10 μM GA_3_ (*n*=48-60). T, trichoblasts; A, atrichoblasts. (B) The presence of 10 μM GA_3_ does not influence the epidermal patterning: the ratio of the epidermal cell lengths of atrichoblasts/trichoblasts is lower in *spy-22* compared with *sec-5* and Col-0. (C) The GL2::4xYFP expression pattern remains largely unchanged in the presence of 10 μM GA_3_. Scale bars: 100 μm. (D) Patterning defects per seedling defined as the number of times an atrichoblast appears in a trichoblast cell file, and vice versa. The average number of patterning events per seedling is higher in *spy-22*, but remained unaffected in the presence of 10 μM GA_3_ in all the lines compared with untreated controls (*n*=16-30). For statistical analysis, one-way ANOVA with Tukey's multiple comparison was carried out (***P≤0.001, *P≤0.05). Data from three biological repeats are shown.

So far, the best-characterized target of SPY is the DELLA protein RGA, which undergoes a conformational change upon O-fucosylation that enhances the interaction with downstream transcription factors, in some cases inhibiting their binding to DNA (Zentella et al., 2017). As a result, *spy* mutants show constitutively active GA signaling. So far, GA signaling has not been described to play a role in epidermal cell patterning in *Arabidopsis thaliana*; hence, we aimed to understand whether the epidermal patterning of *spy-22* was influenced by increased GA signaling. For initial experiments, we treated *spy-22*, *sec-5* and Col-0 with 10 µM GA_3_ and measured the tricho- and atrichoblast cell length in the root meristem transition zone. The distribution pattern remained similar to untreated seedlings, as reported in Fig. 1C. The difference in length of trichoblast cells (13.60±4.21 µm) and atrichoblast cells (16.15±3.38 µm) was smaller in *spy-22* when compared with Col-0 and *sec-5* (Fig. 4A), with a lower atrichoblast/trichoblast ratio (1.3) in *spy-22* also after GA_3_ treatment (Fig. 4B), at a ratio comparable with the untreated seedlings (compare Fig. 1E and 4B). We also observed that the overall root length was not influenced by supplementing additional 2 µM or 10 µM GA_3_. We did not see any effect on the RAM length after growing seedlings on 2 µM and 10 µM GA_3_ supplemented plates for 7 days (Fig. S3). Next, we determined GL2::4xYFP expression in Col-0, *spy-22* and *sec-5* grown on 10 µM GA_3_ and analyzed the cell file patterning in the cell division and transition zones. We quantified this phenotype by counting the number of patterning defects (which we defined as the appearance of atrichoblast cells in trichoblast cell files, and vice versa) per seedling (Fig. 4C). We observed that Col-0 displayed, on average, 1.47 patterning defects per seedling, with 7/19 seedlings showing no patterning defects. After treatment with 10 µM GA_3_, frequencies of patterning defects did not significantly change, with an average of 2 per seedling (Fig. 4D). Similarly, there was no significant change in patterning defects in GL2::4xYFP *sec-5* in untreated controls (2.7 patterning defects per seedling) compared with 10 µM GA_3_-treated seedlings (2.6 patterning defects per seedling) (Fig. 4D). GL2::4xYFP *spy-22* displayed the highest number of patterning defects per seedling (8.1 per seedling) and this did not change significantly upon treatment with 10 µM GA_3_ (7.6 patterning defects per seedling). These results suggest that exogenous application of gibberellin does not influence epidermal patterning in the genotypes analyzed.

**Fig. 5.**
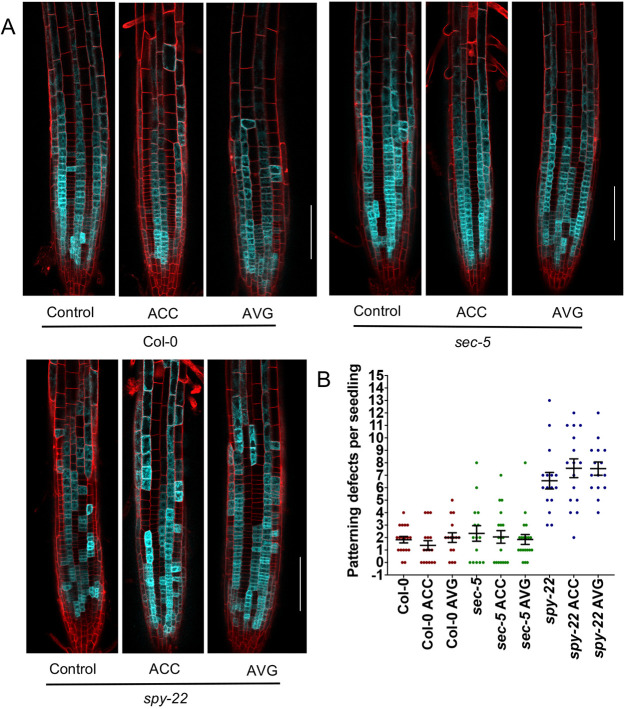
**Ethylene signaling does not regulate GL2 patterning in the root meristem.** (A) GL2::4xYFP expression pattern of 7-day-old seedlings in Col-0, *sec-5* and *spy-22* background in the presence of 1 µM ACC and 100 nM AVG. Scale bars: 100 μm. (B) Patterning defects per seedling defined as the number of times an atrichoblast appears in a trichoblast cell file, and vice versa. The average number of patterning events per seedling remained unaffected in the presence of 1 µM ACC or 100 nM AVG in all the lines compared with untreated controls (*n*=16-20). For statistical analysis, one-way ANOVA with Tukey's multiple comparison was carried out on data from three biological repeats.

The plant hormone ethylene regulates root hair initiation and development (Feng et al., 2017). Treatment of *Arabidopsis thaliana* with ethylene precursor 1-aminocyclopropane-1-carboxylic acid (ACC) induces formation of ectopic root hairs in non-hair positions (Zhang et al., 2016). Moreover, ethylene is known to influence root growth by preventing GA accumulation in roots (Shani et al., 2013). Considering such phytohormonal crosstalk during root development, we explored the possibility of ethylene signaling inducing ectopic epidermal patterning in *spy-22*. GL2::4xYFP seedlings were grown for 7 days on ½ MS plates supplemented with 1 µM ACC or 100 nM aminoethoxyvinylglycine (AVG), a known ethylene biosynthesis inhibitor, before analyzing GL2 expression in Col-0, *spy-22* and *sec-5* (Fig. 5A). We found that the number of patterning defects in all the lines remained unaffected in the presence of ACC and AVG (Fig. 5B). Additionally, we also subjected the EXP7::4xYFP lines in Col-0, *spy-22, sec-5* background to ACC and AVG treatment. We could clearly observe increased root hair length in response to ACC treatment. The appearance of hair cells in non-hair cell files upon ACC treatment and formation of non-hair cells in hair cell files upon AVG treatment, as described by Zhang et al. (2016), was observed at a low frequency (Fig. S4) in Col-0 and *sec-5* backgrounds, while pattering defects in *spy-22* were largely unchanged upon the treatments. These results suggest that ethylene does not affect SPY-dependent regulation of root hair patterning.

**Fig. 6.**
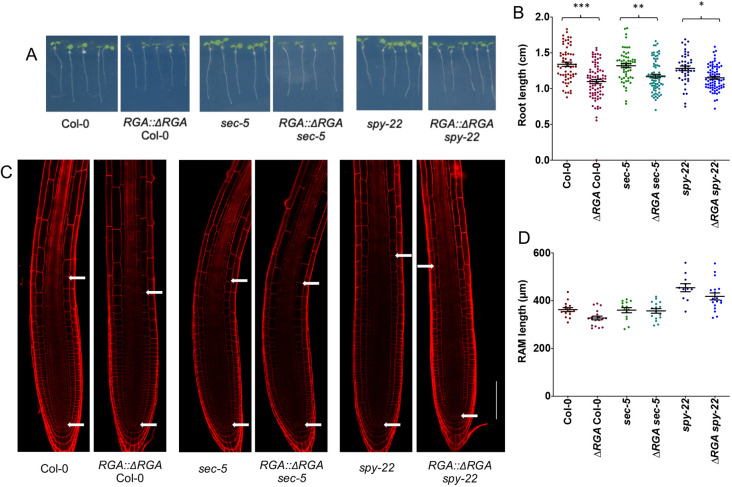
***RGA::ΔRGA* mutants display reduced root length.** (A) 7-day-old seedlings grown on ½ MS plates. (B) *RGA::ΔRGA* Col-0, *RGA::ΔRGA sec-5* and *RGA::ΔRGA spy-22* are significantly shorter than Col-0, *sec-5* and *spy-22,* respectively (*n*=44-79). (C) Longitudinal cross-section images of 7-day-old *RGA::ΔRGA* seedlings mounted in PI. Meristem size was defined as the distance from the quiescent center to the first uppermost cortical cell, which was twice as long as wide, as indicated by white arrows. Scale bar: 100 μm. (D) RAM lengths of 7-day-old seedlings were unaffected by reduced GA signaling in *RGA::ΔRGA* lines (*n*=11-18). For statistical analysis, one-way ANOVA with Tukey's multiple comparison was used (***P≤0.001, **P≤0.01, *P≤0.05). Data from three biological repeats are shown.

Gibberellin signaling in *Arabidopsis thaliana* is regulated via its ability to mediate the degradation of DELLA proteins, a family of growth inhibitors. In the current working model, the degradation of DELLAs de-represses DELLA-interacting proteins, which in turn positively regulate growth (Bao et al., 2020; Davière and Achard, 2016). Most of the available literature on DELLAs is based on work in the L*er*-background. In order to mimic an environment with reduced GA signaling in our mutant lines in Col-0 background, we deleted 17 amino acids of the DELLA domain of RGA as described by Dill et al. (2001), preventing its recognition by the GA receptor GID1. This resulting *RGA::*Δ*RGA* construct was transformed into Col-0, rendering the transformants insensitive to GA and thus constitutively repressing the DELLA-interacting proteins. The resulting plant lines displayed similar phenotypes to those described before in the L*er* background, including smaller leaf and rosette size, darker leaves, and reduced inflorescence axis length (Fig. S5). We then crossed this line into *sec-5* and *spy-22*, in order to test whether reduced GA signaling impacts on root development and root hair patterning. We found that *RGA::*Δ*RGA* Col-0 roots (1.09±0.26 cm) were significantly shorter than Col-0 roots (1.34±0.24 cm). A similar tendency was also seen in *RGA::*Δ*RGA sec-5* roots (1.17±0.21 cm) compared with *sec-5* (1.32±0.21 cm), whereas *RGA::*Δ*RGA spy-22* roots were only slightly shorter (1.15±0.17 cm) compared with *spy-22* (1.28±0.21 cm) (Fig. 6A,B). We did not see any significant difference in the RAM length of *RGA::*Δ*RGA* lines in all backgrounds compared with the Col-0, *spy-22* and *sec-5* parent lines (Fig. 6C,D).

**Fig. 7.**
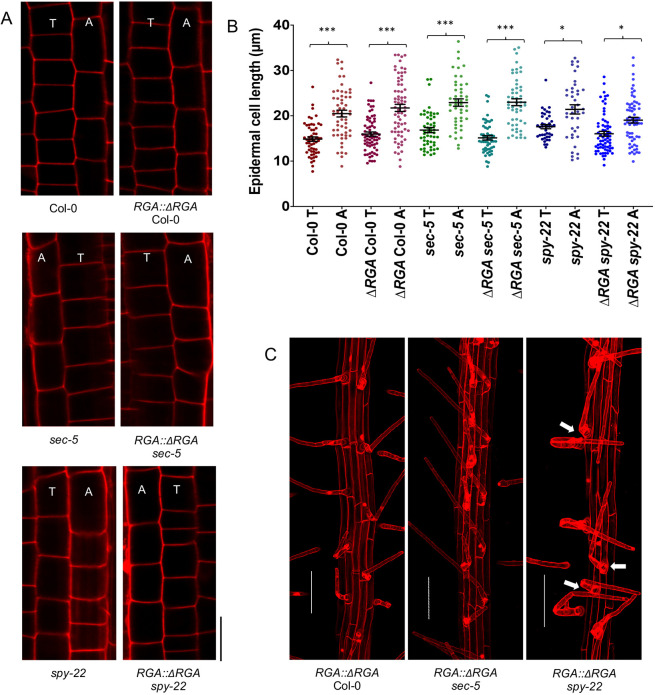
**Reduced GA signaling does not affect epidermal cell patterning or induce ectopic root hairs.** (A) The epidermal layer in the late meristematic region of 7-day-old *RGA::ΔRGA* seedlings mounted in PI. Lengths of four consecutive cells in neighboring (tricho/atrichoblast) files in the late meristem were measured. Scale bar: 20 μm. (B) Atricho- and trichoblast cell lengths from the late meristem of 7-day-old seedlings of *RGA::ΔRGA* Col-0, *RGA::ΔRGA sec-5*, *RGA::ΔRGA spy-22*, Col-0, *sec-5* and *spy-22* (*n*=46-68). (C) 7-day-old *RGA::ΔRGA* Col-0, *RGA::ΔRGA sec-5* and *RGA::ΔRGA spy-22* seedlings grown on ½ MS agar mounted in PI. *RGA::ΔRGA* Col-0 and *RGA::ΔRGA sec-5* did not show ectopic root hairs, while in *RGA::ΔRGA spy-22* ectopic root hair formation was comparable with *spy-22*. Scale bars: 100 μm. For statistical analysis, one-way ANOVA with Tukey's multiple comparison was carried out (***P≤0.001, *P≤0.05). Data from three biological repeats are shown. T, trichoblasts; A, atrichoblasts.

The influence of reduced GA signaling on epidermal tissue patterning in the late meristem was studied by measuring the cell lengths of four consecutive epidermal cells in neighboring cell files (Fig. 7A). There was no significant difference between the cell lengths of atrichoblasts in *RGA::*Δ*RGA* Col-0 (21.73±6.44 µm) and atrichoblasts in Col-0 (20.48±5.59 µm). Likewise, trichoblast cells in *RGA::*Δ*RGA* Col-0 (15.97±3.83 µm) were similar in length to Col-0 trichoblasts (14.85±3.59 µm) (Fig. 7B). The ratio of atrichoblast/trichoblast cell length in *RGA::*Δ*RGA* Col-0 (1.40) was thus unchanged compared with Col-0 (1.44) (Fig. S6). We found similar results when comparing cell lengths of *RGA::*Δ*RGA sec-5* and *sec-5*, as well as *RGA::*Δ*RGA spy-22* and *spy-22* (Fig. 7B). The ratio of atrichoblast/trichoblast in the case of *RGA::*Δ*RGA spy-22* (1.23) was lower, as seen in the *spy-22* parent line (1.21) (Fig. S6).

Examination of *RGA::*Δ*RGA* Col-0 and *RGA::*Δ*RGA sec-5* roots demonstrated that root hair patterning is similar to that of Col-0 and sec-5, respectively, showing no discernible ectopic root hair formation, and *RGA::*Δ*RGA spy-22* still displayed ectopic root hairs resembling the parent line *spy-22* (Fig. 7C). To further assess our findings regarding GA-dependent root development, we analyzed the phenotypes of the GA biosynthesis mutant *ga1-4* and the *della* quintuple mutant, *gai-t6 rga-t2 rgl1-1 rgl2-1 rgl3-1*, both in L*er*. At 7 days, *ga1-4* showed a shorter RAM, whereas the *della* quintuple mutant displayed a RAM size similar to that of L*er* wild type (Fig. S7A,B). The reduced RAM of *ga1-4* was complemented by a reduced overall root length (Fig. S7C). To further analyze the involvement of GA in epidermal patterning, atrichoblast and trichoblast cell lengths in the late meristem were measured (Fig. S7D). We found that atrichoblasts were significantly longer than trichoblasts in all three lines (Fig. S7E), as seen in Col-0 and *sec-5*, but not in *spy-22* (Fig. 1D,E). The *della* quintuple mutant shows increased GA signaling, as observed in *spy-22.* However, *della* mutants did not display ectopic root hair formation, as seen in *spy-22.* In addition, the GA-deficient *ga1-4* showed regular root hair patterning without ectopic hairs, similar to L*er* wild type (Fig. S7F). Taken together, we did not find evidence that epidermal cell patterning defects in *spy-22* are dependent on GA signaling.

## DISCUSSION

**Fig. 8.**
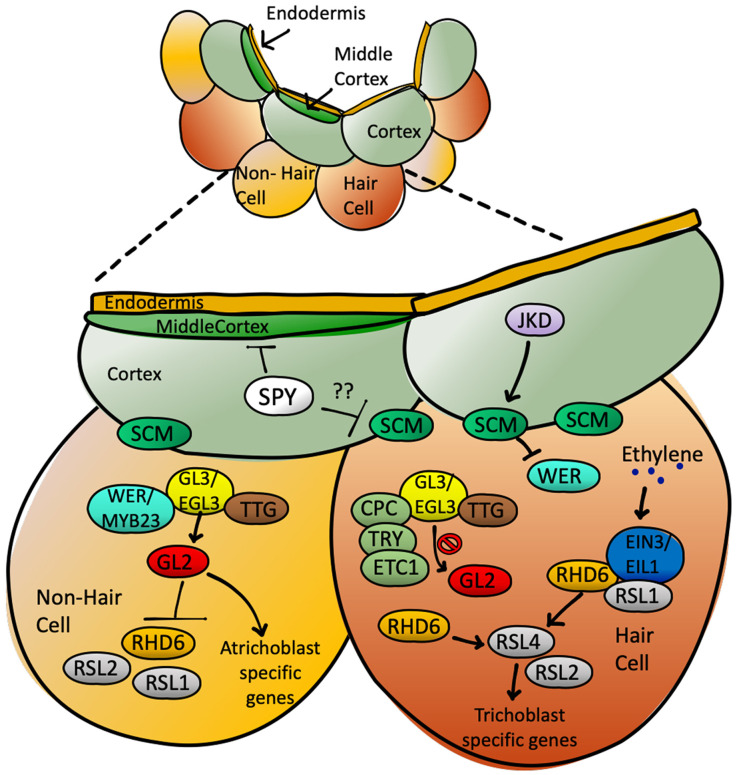
**SPY regulates epidermal cell patterning upstream of WER.** Various fate determination factors are responsible for imparting epidermal patterning and tissue organization in the *Arabidopsis thaliana* roots. Non-hair cell fate is largely modulated by WER and GL2. *spy-22* mutants display defects in epidermis and subsequent root hair patterning by regulating cell fate determination upstream of WER.

Root hairs are essential for the uptake of water and nutrients, as they can sense nutrients in the soil and react by increasing the root surface in a very flexible way. Root hair patterning is therefore regulated by internal as well as environmental factors, allowing for a high degree of plasticity in the developmental program. Thus, many different pathways feed into the regulation of cell fate determination in the epidermis, including a number of hormones such as auxin, ethylene and brassinosteroids (Balcerowicz et al., 2015; Borassi et al., 2020; Kuppusamy et al., 2009; Liu et al., 2018; Shibata and Sugimoto, 2019). Root hair patterning in *Arabidopsis thaliana* has been studied extensively and represents a very useful model system for analysis of plasticity in cell fate determination. In recent years, a number of tools have been made available to monitor the establishment of hair- and non-hair cell files in the root apical meristem, including a set of transcriptional reporters labeling specific cell types (Marquès-Bueno et al., 2016). Here, we present evidence that O-fucosylation is involved in establishing root hair cell patterning. Using a number of transcriptional reporters, genetics and phenotypical analysis, we show that root hair cell patterning is impaired in the O-fucosyltransferase mutant *spy-22*. Monitoring the expression of WER by using a transcriptional reporter suggests that the patterning defect in *spy-22* is established already early on during epidermal cell fate determination, potentially due to defects in cortex development or cell-to-cell communication between cortex and epidermis, as these processes regulate cell type-specific WER expression levels (Fig. 8). The atypical receptor-like kinase SCRAMBLED (SCM) plays an important role in signaling from the cortex to the epidermis and further on to WER in this context (Gao et al., 2019; Kwak et al., 2005). Further experiments targeting the function, localization or turnover of SCM might help determine how SPY participates in cell-to-cell communication at this stage, or alternatively in upstream signaling events in the cortex. Other potential targets of SPY include the transcription factor JACKDAW (JKD), which is expressed in the cortex and regulates epidermal cell fate in a non-cell autonomous way, or other regulators of SCM, such as QKY (Hassan et al., 2010; Song et al., 2019).

Post-translational modification by attachment of O-fucose or O-GlcNAc is still not very well understood in plants. The best-studied target is the gibberellin signaling repressor RGA, where O-GlcNAc and O-fucose have opposite effects on its activity, probably by inducing conformational changes (Zentella et al., 2016, 2017). Accordingly, *spy* mutants show many phenotypes that can be associated with gibberellin signaling, such as paclobutrazol resistance, early flowering or elongated growth (Olszewski et al., 2010; Silverstone et al., 2007). *spy-5* showed an increase in trichome formation (Perazza et al., 1998; Silverstone et al., 2007), which has also been linked to constitutive GA signaling, as trichome initiation is positively regulated by GA (Chien and Sussex, 1996; Kim et al., 2018). Trichome and root hair formation share a similar genetic regulatory network with some of the genes involved being active both in leaf and root epidermis (Ishida et al., 2008). In our study, we did not find an indication that consequences of altered O-fucosylation on root epidermal patterning would require gibberellin signaling, as exogenous application of GA did not affect root hair patterning (Fig. 4). Ethylene not only regulates root hair initiation but also controls root growth by inhibiting GA accumulation by stabilizing the DELLA proteins (Achard et al., 2003; Shani et al., 2013). We observed that ectopic GL2 patterning of *spy-22* mutants was independent of ethylene signaling (Fig. 5). *RGA::*Δ*RGA* lines with impaired GA signaling consistently produced shorter roots compared with parent backgrounds (Fig. 6A,B). RGA regulates GA signaling in the elongation zone (Shani et al., 2013), hence altered RGA activity in the elongation zone could be responsible for the reduced root lengths. We did not observe root hair patterning defects in *RGA::*Δ*RGA* lines, whereas *RGA::*Δ*RGA spy-22* still displayed patterning defects similar to *spy-22* (Fig. 7C). Further analysis of the GA-deficient mutant *ga1-4* and *della* quintuple mutants with upregulated GA signaling, revealed that none of these lines displayed abnormal root hair patterning (Fig. S7F). These observations further confirm that ectopic root hair formation in *spy-22* mutants is indeed independent of GA signaling. The observed increase in cell numbers of *spy-22* meristems (Fig. S1) is probably independent of the patterning defect, but further studies are necessary to address whether this increased cell division is dependent on GA-signaling.

Overall, we suggest a model whereby SPY regulates root hair cell fate determination by affecting the spatial order of WER expression, which then signals down to patchy expression of GL2 and EXP7, leading to ectopic root hair formation (Fig. 2). Thus, O-glycosylation potentially regulates the function of upstream regulators such as SCM or the cell-to-cell communication from cortex to the epidermis (Fig. 8), but further studies are necessary to reveal the direct targets of SPY in this context.

## MATERIALS AND METHODS

### Plant material and growth conditions

All mutant lines used in this study were obtained from the Nottingham Arabidopsis Stock Centre NASC. Col-0 and L*er* ecotype of *Arabidopsis thaliana* were referred to as wild-type controls. T-DNA insertion lines of *spy-22* (SALK_090582) and *sec-5* (SALK_034290), EMS-mutant *wer-1* (N6349) and previously published reporter lines WER::4xYFP (N2106117), GL2::4xYFP (N2106121) and EXP7::4xYFP (N2106118) (Marquès-Bueno et al., 2016) in Col-0 background, as well as *ga1-4* (N3105) and the *della* quintuple mutant *gai-t6 rga-t2 rgl1-1 rgl2-1 rgl3-1* (N16298), both in L*er* background, were used. After surface sterilization with 70% ethanol, the seeds were plated onto ½ Murashige and Skoog medium [2.15 g/l MS Salts, 0.25 g/l MES (pH 5.7), 1% agar]. After stratification in the dark at 4°C for 2 days, they were vertically grown in long-day conditions (16 h light/8 h dark) at 22°C.

### Hormone treatments

For GA treatment, seeds were surface sterilized with 70% ethanol and transferred to ½ MS medium containing 2 µM or 10 µM GA_3_ (for root and RAM length measurements) and 10 µM GA_3_ (for patterning experiments), stratified in the dark at 4°C for 2 days and vertically grown in long-day conditions (16 h light/8 h dark) at 22°C for 7 days.

For ethylene treatments, seeds were surface sterilized with 70% ethanol and transferred to ½ MS medium containing 1 µM ACC or 100 nM AVG, stratified in the dark at 4°C for 2 days and vertically grown in long-day conditions (16 h light/8 h dark) at 22°C for 7 days.

For experiments involving *ga1-4*, all lines were surface sterilized with 70% ethanol and treated with 10 µM GA_3_ for 7 days at 4°C in dark to enable germination. Subsequently, the seeds were thoroughly rinsed with sterile distilled water, transferred to ½ MS medium and vertically grown in long-day conditions (16 h light/8 h dark) at 22°C for 7 days.

### Microscopy

For imaging, a Leica TCS SP5 confocal microscope with an HCX PL APO CS 20.0×0.70 IMM UV objective was used. Seedlings were mounted in propidium iodide (PI) (0.02 mg/ml) for staining the cell wall prior to imaging. A DPSS561 laser was used to excite PI at 561 nm (emission 584-735 nm with standard PMT), and an Argon Laser at 30% intensity was used to excite YFP at 514 nm (emission 524-552 with HyD detector). Z stacks were taken for visualizing root hairs and maximum projections were made using the Leica LAS AF lite software.

### Phenotyping and image quantification

Measurements and quantifications were performed using the LAS×Leica Software. For studying the RAM length, seedlings were mounted in PI (0.02 mg/ml). We measured the distance from quiescent center until the uppermost first cortical cell, which was twice as long as wide as described by Feraru et al. (2019). For epidermal cell patterning, lengths of four consecutive epidermal cells from neighboring (tricho/atrichoblast) files in the late meristem were measured (Löfke et al., 2015). For analyzing the patterning frequency in GL2::4xYFP, we checked for its expression in cell division and transition zones. We defined the occurrence of trichoblast cells in an atrichoblast cell file, and vice versa, as a patterning defect and counted the number of such patterning events in each seedling. For root length measurements, ½ MS plates with seedlings were scanned using Epson Perfection V700 scanner and the root lengths were measured using ImageJ.

### Data analysis

We used GraphPad Prism 8 for generating graphs. Error bars in graphs indicate s.e.m. One-way ANOVA and Tukey's multiple comparison test or Student's *t*-test were performed for statistical analysis of the data. Sample sizes (*n*) for all experiments are given in the respective figure legends.

### Plasmid construction and generation of transgenic lines

To generate a GA-insensitive, stabilized version of RGA in the Col-0 background, *RGA::*Δ*RGA* was amplified from genomic DNA of Col-0 using Q5 high-fidelity DNA polymerase (NEB). Two overlapping fragments lacking 17 amino acids covering the DELLA domain, as described previously by Feng et al. (2008) were generated using the following primer pairs: #270 (5′-tacaaaaaagcaggctccactagtactaattattcgtctgtc-3′) and #272 (5′-gttcgagtttcaaagcaacctcgtccatgttacctccaccgtc-3′), #273 (5′-gacggtggaggtaacatggacgaggttgctttgaaactcgaac-3′) and #271 (5′-gctgggtctagatatctcgagtacgccgccgtcgagag-3′). The resulting overlapping fragments were then cloned into a Gateway pENTR4 vector backbone linearized with NcoI/XhoI using Gibson Assembly (NEB). The assembled plasmid was transformed into electrocompetent DH10b *E. coli* cells; positive clones were selected on LB medium using kanamycin (50 µg/ml) and confirmed by sequencing. Confirmed entry clones were digested with AsiI to destroy the kanamycin resistance of the pENTR4-backbone, and recombined with pEarleyGate303 (Earley et al., 2006) using Gateway LR Clonase ll enzyme mix to generate a plant expression vector. Positive colonies were selected for kanamycin (50 µg/ml) resistance, confirmed plasmids were electro-transformed into *Agrobacterium tumefaciens* GV3101 and used for transforming *Arabidopsis thaliana* ecotype Col-0 by floral dipping (Clough and Bent, 1998). Stable transformants with a strong GA-deficient phenotype were selected before crossing with *spy-22* and *sec-5*.

## Supplementary Material

Supplementary information

Reviewer comments
