# Supplementary material for: The Arabidopsis O-fucosyltransferase SPINDLY regulates root hair patterning independently of gibberellin signaling
Source: Development. 2020 Oct 9;147(19):dev192039. doi: 10.1242/dev.192039 (PMC7567127; doi:10.1242/dev.192039)
Supplement: Supplementary information [file develop-147-192039-s1.pdf]

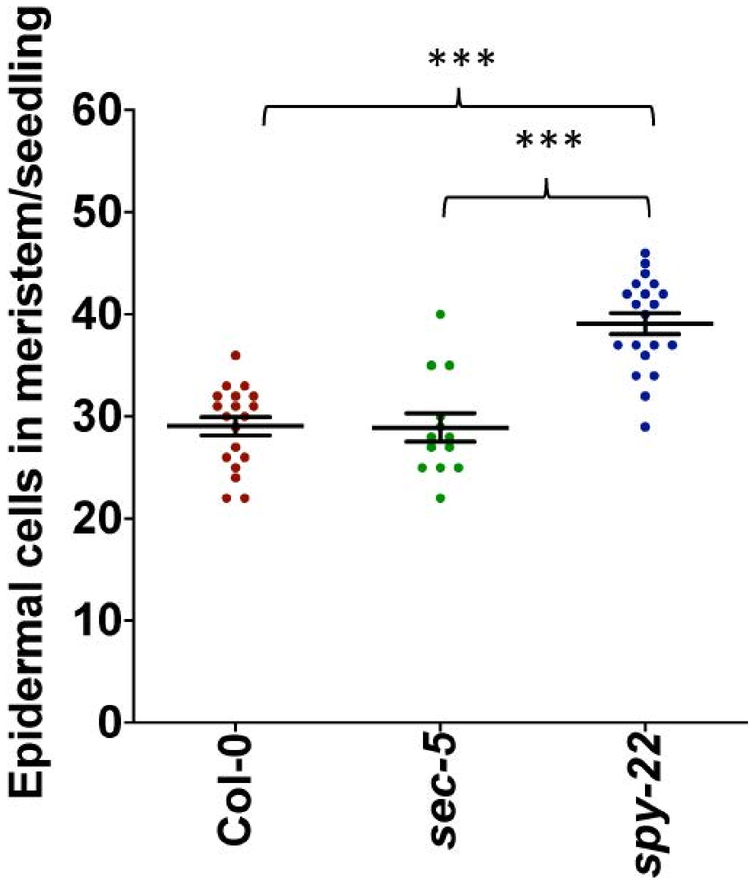

Fig. S1. *spy-22* mutants have an increased number of cells in the RAM. Number of epidermal cells in the meristem of 7-day old O-glycosylation mutants. Meristems of *spy-22* have a higher number of epidermal cells (39.10 +/- 4.599) compared to Col-0 (29.05 +/- 3.965) and *sec-5* (28.92 +/- 5.008). For statistical analysis, One-way ANOVA with Tukey's multiple comparison was done (*** P ≤ 0.001), data from three independent biological repeats is shown.

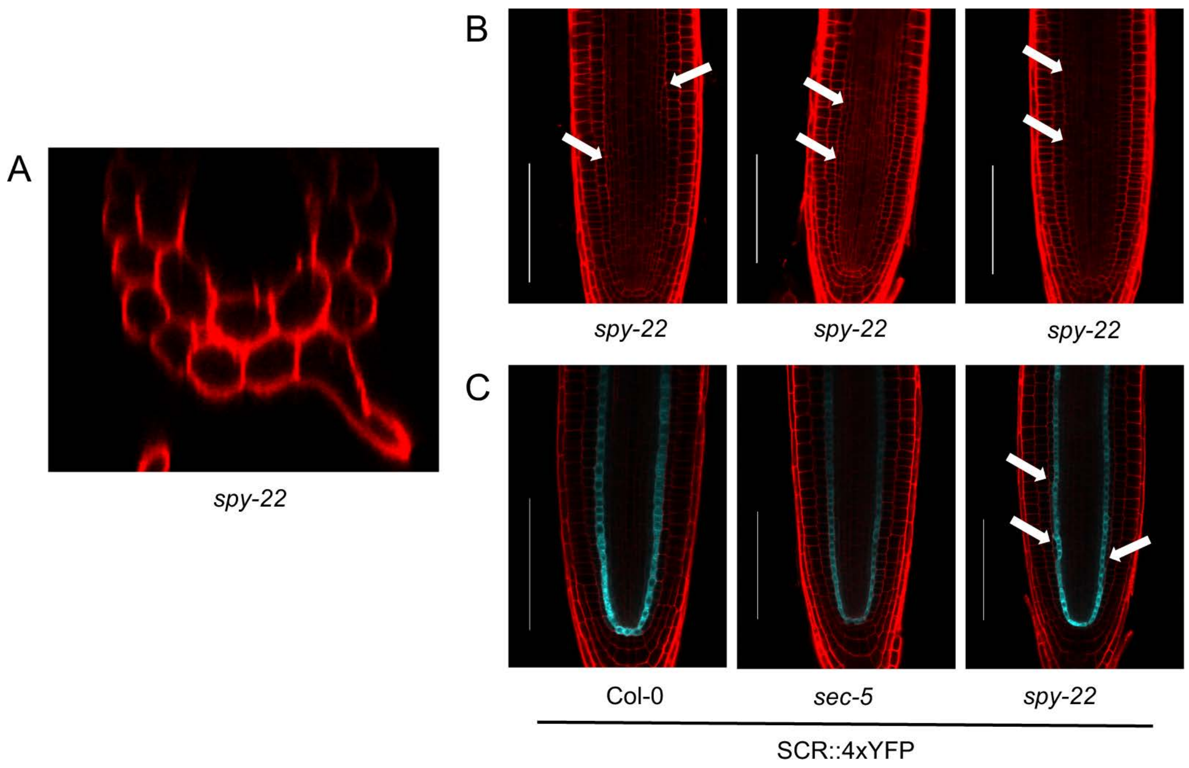

Fig. S2. ***spy-22*** **shows abnormal hair cell positioning with respect to underlying cortex. A**- Transverse section of the fully elongated zone in 7-day old *spy-22* seedlings. Ectopic root hair cells are adhered to only a single underlying cortex cell in *spy-22*. **B**- 7-day old *spy-22* seedlings, arrows indicate middle cortex formation. This extra layer of cortex is formed between cortex and endodermis and has been previously described by Cui et al. 2014 in other *spy* alleles. scale bar – 100 µm. **C**- SCR::4xYFP expression in Col-0, *sec-5* and *spy-22* is restricted to the endodermis. The middle cortex proliferation in *spy-22* is unique and independent of SCR expression in the endodermis.

Development • Supplementary information

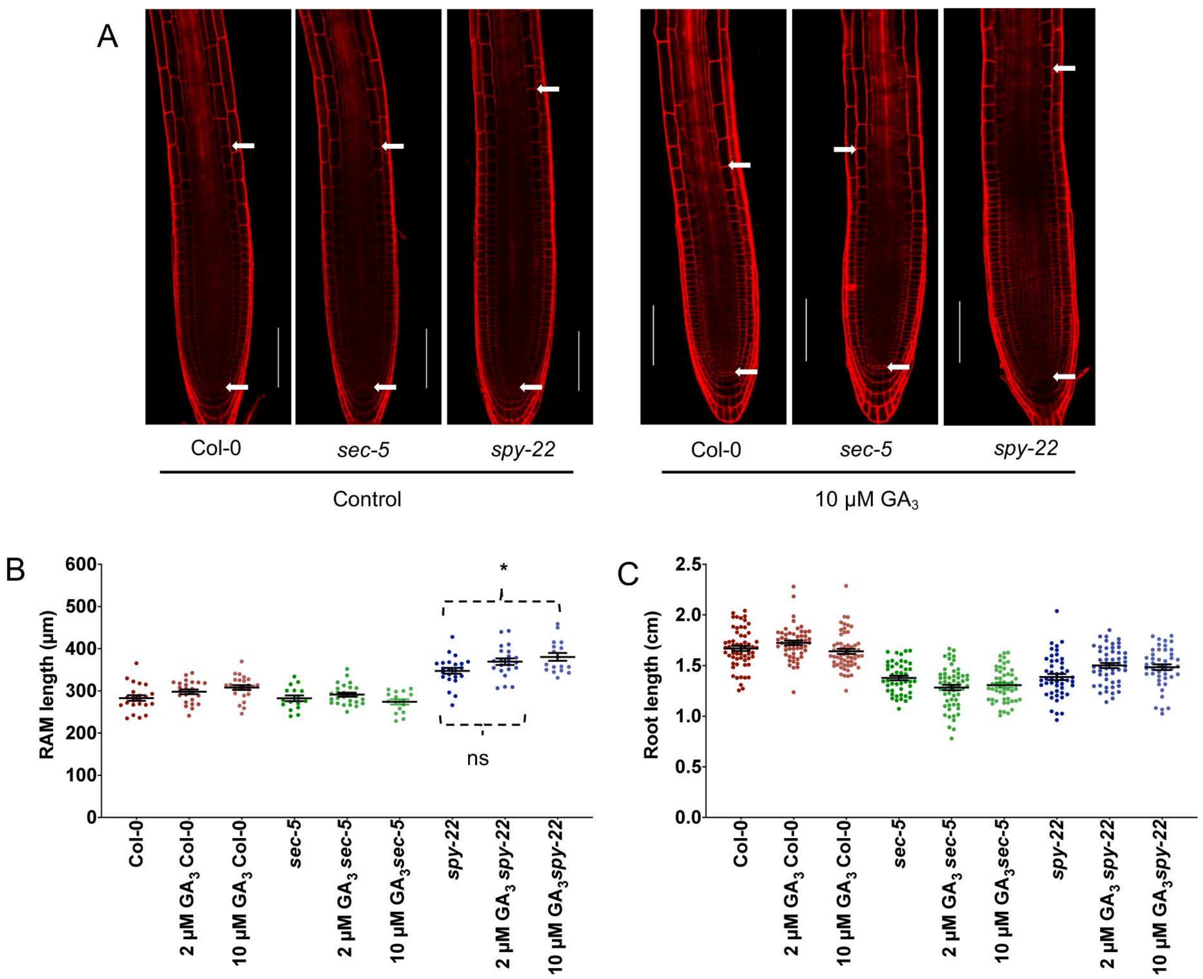

Fig. S3. RAM length is unaffected by exogenous GA treatment. A- Longitudinal cross section images of 7-day old seedlings grown in ½ MS plates supplemented with 10 μM GA_3. Meristem size was defined as the distance from the quiescent center to first uppermost cortical cell which was twice as long as wide, as indicated by white arrows, scale bar – 100 μm. **B**- Addition of 2 μM and 10 μM GA_3 did not alter RAM length. n = 16-25. **C**- Length of 7-day old seedlings grown on ½ MS plates with 2 μM and 10 μM GA_3. n = 45-60. For statistical analysis, One-way ANOVA with Tukey's multiple comparison was done (* $P \leq 0.05$), data from three independent biological repeats is shown.

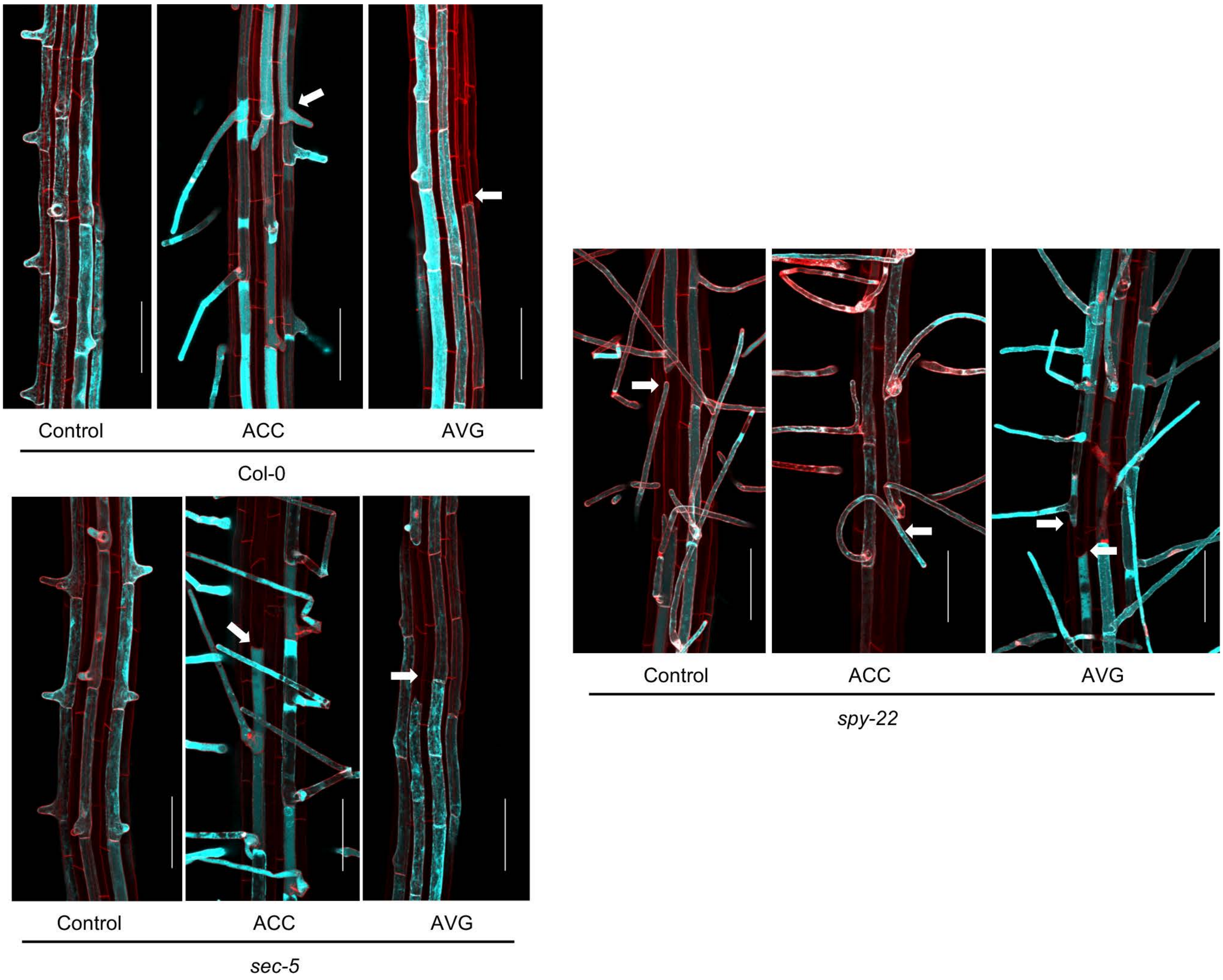

Fig. S4. Ethylene signaling does not regulate ectopic hair formation in *spy-22.* A- 7-day old EXP7::4xYFP seedlings in Col-0, *sec-5* and *spy-22* background grown on ½ MS plates supplemented with 1 µM ACC and 100 nM AVG. ACC treatment induced formation longer root hairs in Col-0 and *sec-5*. Additionally, ACC led to occurrence of hair cells in non-hair cell files in Col-0 and *sec-5* background in a very low frequency, and treatment with AVG very slightly induced the formation of non-hair cells in a hair cell file in Col-0 and *sec-5* background. However, the ectopic root hair formation and root hair length were evidently unaffected by ACC and AVG treatment in *spy-22*. Scale bar – 100 µM . Representative pictures of three biological repeats are shown.

Development • Supplementary information

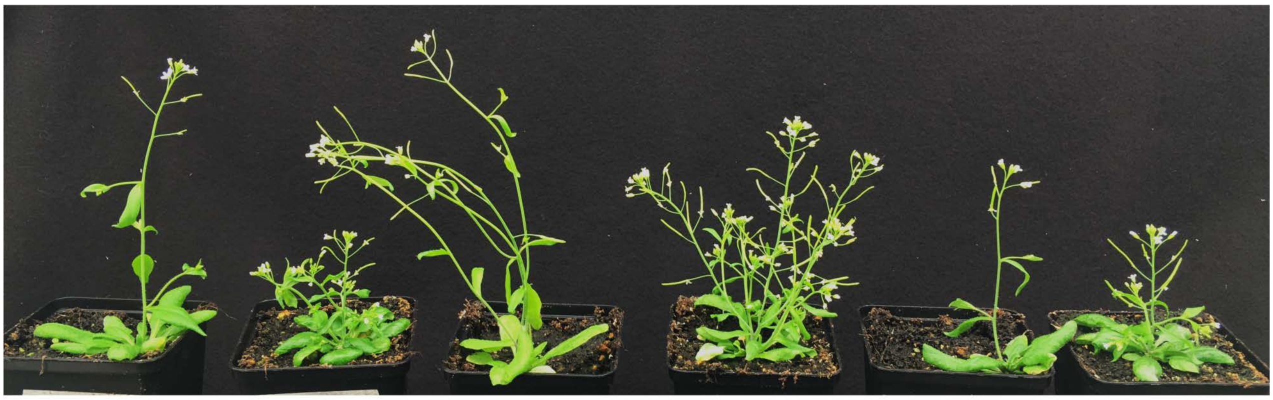

Fig. S5. Rosette and shoot phenotype of *RGA::ΔRGA* mutants. Col-0, *spy-22*, *sec-5* and their crosses with *RGA::ΔRGA* Col-0, a line expressing a stabilized version of the GA-signaling repressing DELLA protein RGA. All *RGA::ΔRGA* lines show phenotypes characteristic for low GA signaling, like smaller rosette size and shorter inflorescences.

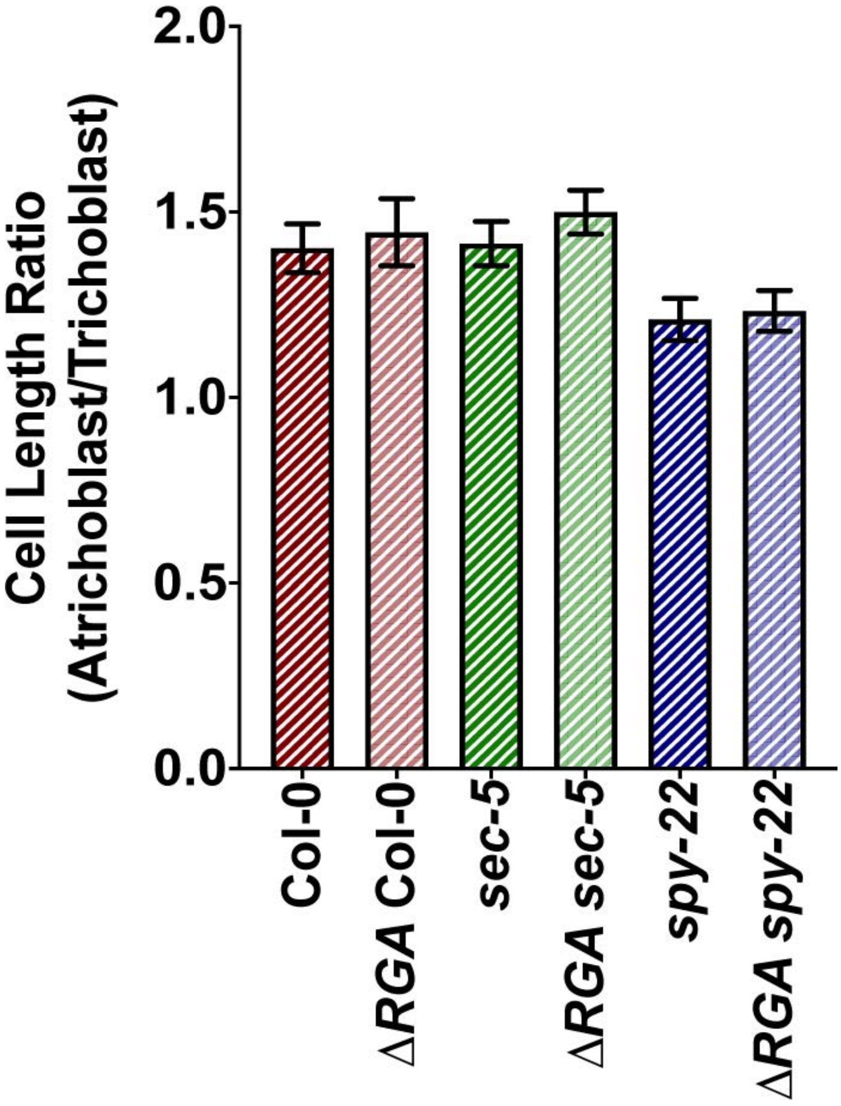

Fig. S6. Atrichoblast/trichoblast cell length ratio of *RGA::ΔRGA* mutants. The ratio of atrichoblast/trichoblast cell lengths in *RGA::ΔRGA* Col-0 (1.40) was similar to Col-0 (1.44). Compared to all lines, the ratio of *RGA::ΔRGA spy-22* (1.23) was lower, similar to *spy-22* (1.21).

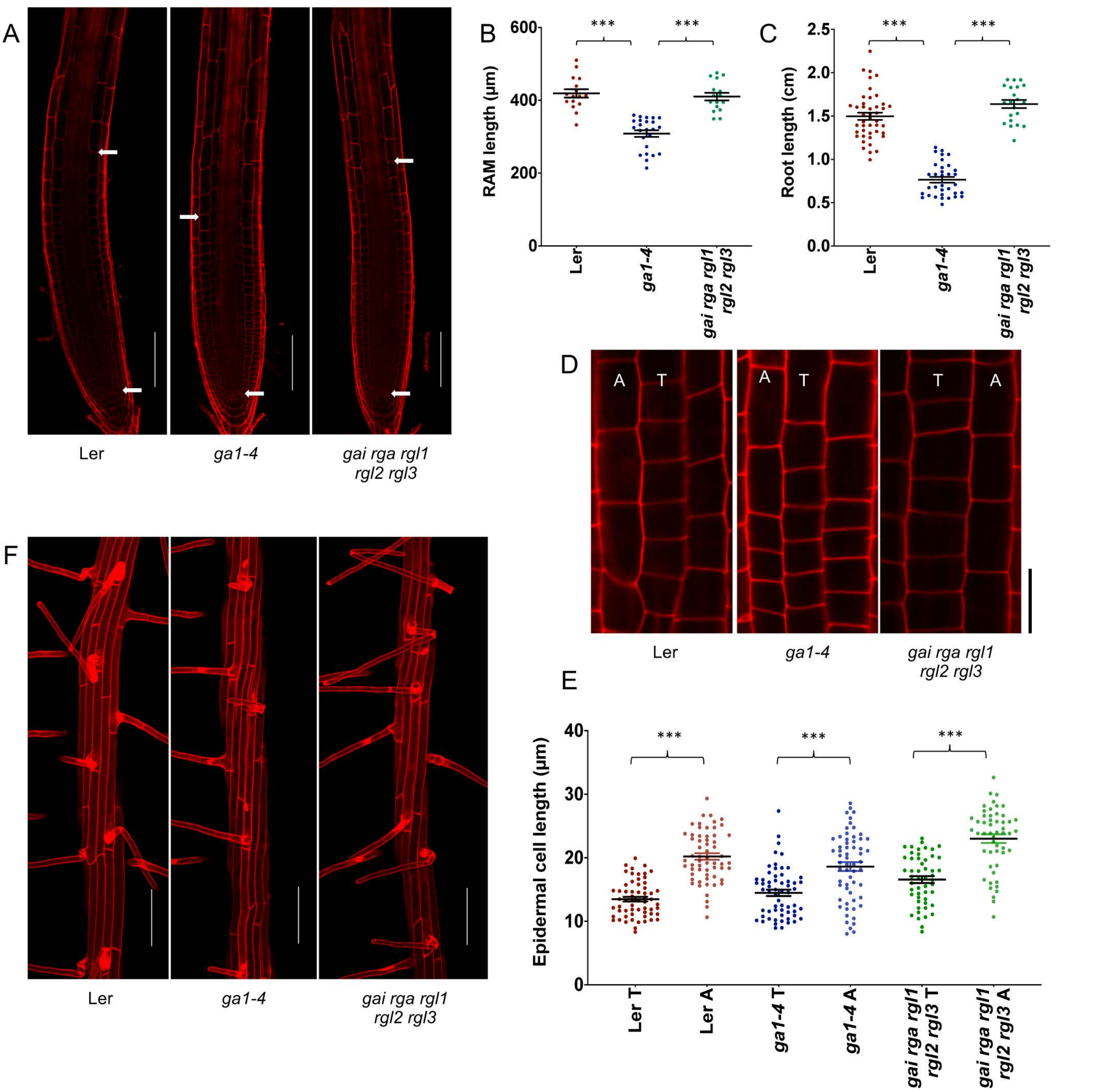

Fig. S7. GA deficient *ga1-4* and *della* mutants with high GA signaling do not display ectopic root hairs. A- Longitudinal cross section of 7-day old L*er*, *ga1-4* and *gai rga rgl1 rgl2 rgl3* seedlings, meristem size was defined as the distance from the quiescent center to first uppermost cortical cell which was twice as long as wide, as indicated by white arrows, scale bar – 100 µm. **B-** 7 day old seedling of *ga1-4* display significantly shorter RAM compared to L*er* and *gai rga rgl1 rgl2 rgl3.* n = 16-24. **C-** The overall root length of 7-day old *ga1-4* seedlings is significantly shorter than L*er* and *gai rga rgl1rgl2 rgl3.* n = 21-44. **D-** The epidermal layer in the late meristematic region of 7-day old L*er*, *ga1-4* and *gai rga rgl1 rgl2 rgl3* seedlings. Lengths of 4 consecutive cells in neighbouring (tricho/atrichoblast) files in the late meristem were measured, scale bar – 20 µm. **E-** The atrichoblast cells are significantly longer than the trichoblast cells in all the lines. n = 51-60. **F-** 7-day old seedlings of L*er*, *ga1-4* and *gai rga rgl1 rgl2 rgl3* do not display ectopic root hair formation in the elongated zone. For statistical analysis, One-way ANOVA with Tukey's multiple comparison was done (*** P ≤ 0.001), data from three independent biological repeats is shown.
