# Supplementary material for: The Arabidopsis O-fucosyltransferase SPINDLY regulates root hair patterning independently of gibberellin signaling
Source: Development. 2020 Oct 9;147(19):dev192039. doi: 10.1242/dev.192039 (PMC7567127; doi:10.1242/dev.192039)
Supplement: Reviewer comments [file develop-147-192039_review_history.pdf]

The *Arabidopsis* O-fucosyltransferase SPINDLY regulates root hair patterning independently of gibberellin signalling

Krishna Vasant Mutanwad, Isabella Zangl and Doris Lucyshyn
DOI: 10.1242/dev.192039

Editor: Yka Helariutta

Review timeline
Original submission: 23 April 2020
Editorial decision: 8 June 2020
First revision received: 4 August 2020
Accepted: 7 September 2020

Original submission

First decision letter

MS ID#: DEVELOP/2020/192039

MS TITLE: Arabidopsis O-fucosyltransferase SPINDLY regulates root hair patterning independently of gibberellin signalling

AUTHORS: Krishna Vasant Mutanwad, Isabella Zangl, and Doris Lucyshyn

I have now received all the referees' reports on the above manuscript, and have reached a decision. The referees' comments are appended below, or you can access them online: please go to BenchPress and click on the 'Manuscripts with Decisions' queue in the Author Area.

As you will see, the referees express considerable interest in your work, but have some significant criticisms and recommend a substantial revision of your manuscript before we can consider publication. If you are able to revise the manuscript along the lines suggested, which may involve further experiments, I will be happy receive a revised version of the manuscript. Your revised paper will be re-reviewed by one or more of the original referees, and acceptance of your manuscript will depend on your addressing satisfactorily the reviewers' major concerns. Please also note that Development will normally permit only one round of major revision.

We are aware that you may currently be unable to access the lab to undertake experimental revisions. If it would be helpful, we encourage you to contact us to discuss your revision in greater detail. Please send us a point-by-point response indicating where you are able to address concerns raised (either experimentally or by changes to the text) and where you will not be able to do so within the normal timeframe of a revision. We will then provide further guidance. Please also note that we are happy to extend revision timeframes as necessary.

Please attend to all of the reviewers' comments and ensure that you clearly highlight all changes made in the revised manuscript. Please avoid using 'Tracked changes' in Word files as these are lost in PDF conversion. I should be grateful if you would also provide a point-by-point response detailing how you have dealt with the points raised by the reviewers in the 'Response to Reviewers' box. If you do not agree with any of their criticisms or suggestions please explain clearly why this is so.

© 2020. Published by The Company of Biologists under the terms of the Creative Commons Attribution License (https://creativecommons.org/licenses/by/4.0/).

Reviewer 1

Advance summary and potential significance to field

The manuscript by Mutanwad et al nicely identifies a GA independent role of SPY in root hair patterning. The paper is well written and easy to understand.

Comments for the author

I do not have major comments but the connection between SPY and WER or GL2 is based primarily on aberrant expression of these reporters in spy mutant bacjground. I would normally expect this to be complemented by genetics e. g. spy/gl2 and spy/wer double mutants. In additon to the genetic factors, plant hormone ethylene plays a key role in root hair patterning. Although spy has GA independent role, I would appreciate if authors could comment on whether alterede patterning in spy could be due to activation of ethylene response. this could be easily achieved either genetically or by treatment with ethylene signaling biosynthesis inhibitors.

Reviewer 2

Advance summary and potential significance to field

The manuscript is concerned with the influence of protein glycosylation on root hair formation in Arabidopsis. The authors show that loss of function of the protein O-fucosyltransferase SPINDLY (SPY) results in altered root epidermal cell patterning with ectopic root hair formation. Through examining the effect of the mutation on the expression patterns of transcription factors that determine cell specification, they conclude that SPY acts upstream of GLABRA2 (GL2) and WEREWOLF that regulate formation of non-hair epidermal cells. SPY and the O-linked N-acetylglucosaminetransferase SECRET AGENT (SEC) are known to respectively enhance or suppress the activity of DELLA proteins, which are components of the gibberellin (GA) signalling pathway. The authors show that loss of SEC function has no effect on root hair formation, and present evidence indicating that the action of SPY to regulate root hair formation is independent of GA signalling. These are potentially novel findings that would contribute to understanding the regulation of cell patterning in the Arabidopsis root.

Comments for the author

It is highly likely that SPY has protein substrates other than DELLAs and it is reasonable to suppose that it can function in other signalling pathways. It is noteworthy that trichome formation, which involves some of the same signalling components as root-hair formation, such as GL2 and ZFP5, is influenced by GA signalling. This seems relevant to the discussion, but is not mentioned. The manuscript provides no clues as to the substrate of SPY in epidermal cell patterning and there is clearly more work required.

While the conclusions are reasonable, I thought that the evidence for non-involvement of GA signalling in SPY-regulated root hair formation is a little thin. Root development is quite tolerant of changes in GA status. GA levels are close to saturating for root growth, while GA content must be reduced to very low levels before it becomes limiting for root growth. The authors showed no effect of GA application on root hair formation in the wild-type or on the altered cell patterning in the spy-22 mutant. Did they see other changes in root development? In order to suppress GA signalling, they expressed a mutant form of the DELLA protein RGA containing a 17-amino acid deletion, rendering it insensitive to GA-induced degradation. The mutant RGA did not affect root hair formation in Col-0 or spy-22, but what about other aspects of root morphology?
It is unclear whether the increased RGA content in this plant would suppress GA signalling sufficiently. It is also worth considering the potential for the deletion in the mutant RGA altering how it is modified by SPY. It would be worth including severe GA-deficient mutants, which are known to have reduced meristem and final cell size in the root.

Other minor points:
Line 36: RGA is an abbreviation for REPRESSOR OF ga1-3.

© 2020. Published by The Company of Biologists under the terms of the Creative Commons Attribution License (https://creativecommons.org/licenses/by/4.0/).

Line 149: DELLAs can act as transcription co-activators as well as suppressors.
Activation of RGA by SPY has the potential to promote gene expression in some physiological contexts.
Lines 173-174: It is not clear whether all growth suppression by DELLAs is due directly to reduced gene expression.
Line 178: The GA receptor is GID1. It should be differentiated from GID2 which, in rice, is the F-box component of the E3 ligase involved in DELLA ubiquitination.

First revision

Author response to reviewers' comments

We thank the reviewers for the evaluation of our manuscript and their suggestions for improvement. We have added new data and modified our text and figures to address their concerns as detailed below. Our responses are marked in blue.

Reviewer 1 Advance Summary and Potential Significance to Field:
The manuscript by Mutanwad et al nicely identifies a GA independent role of SPY in root hair patterning. The paper is well written and easy to understand.

Reviewer 1 Comments for the Author:
I do not have major comments but the connection between SPY and WER or GL2 is based primarily on aberrant expression of these reporters in spy mutant bacjground. I would normally expect this to be complemented by genetics e. g. spy/gl2 and spy/wer double mutants. In additon to the genetic factors, plant hormone ethylene plays a key role in root hair patterning. Although spy has GA independent role, I would appreciate if authors could comment on whether alterede patterning in spy could be due to activation of ethylene response. this could be easily achieved either genetically or by treatment with ethylene signaling biosynthesis inhibitors.

We thank the reviewer for this overall positive response. We agree with the points raised, and have added the following data in the revised manuscript:

Fig. 3 shows confocal images of a *spy-22 wer-1* cross, with a description in line 180 - 182. Unfortunately, we were not able to obtain a *gl2* mutant in Col-0-background and generate a cross with *spy-22* within the time-frame of this revision.

Fig. 5 and Fig. S4 show the GL2::4xYFP- and EXP7::4xYFP- expressing lines, respectively, after ACC and AVG treatments, with the data described in line 225 - 241. In summary, on the level of GL2-expression, we did not see a similar effect of ACC or AVG on patterning like seen in *spy-22* (Fig. 5). We could observe weak effects of ACC and AVG downstream of GL2 (using the EXP7::4xYFP-lines) like described in the literature before (Zhang et al. 2016), but overall, they were much weaker than the patterning defect seen in *spy-22* (Fig. S4). We have also updated the working model incorporating these results (now Fig. 8).

Reviewer 2 Advance Summary and Potential Significance to Field:
The manuscript is concerned with the influence of protein glycosylation on root hair formation in Arabidopsis. The authors show that loss of function of the protein O-fucosyltransferase SPINDLY (SPY) results in altered root epidermal cell patterning with ectopic root hair formation. Through examining the effect of the mutation on the expression patterns of transcription factors that determine cell specification, they conclude that SPY acts upstream of GLABRA2 (GL2) and WEREWOLF that regulate formation of non-hair epidermal cells. SPY and the O-linked N-acetylglucosaminetransferase SECRET AGENT (SEC) are known to respectively enhance or suppress the activity of DELLA proteins, which are components of the gibberellin (GA) signalling pathway. The authors show that loss of SEC function has no effect on root hair formation, and present evidence indicating that the action of SPY to regulate root hair formation is independent of GA

© 2020. Published by The Company of Biologists under the terms of the Creative Commons Attribution License (https://creativecommons.org/licenses/by/4.0/).

signalling. These are potentially novel findings that would contribute to understanding the regulation of cell patterning in the Arabidopsis root.

Reviewer 2 Comments for the Author:

It is highly likely that SPY has protein substrates other than DELLAs and it is reasonable to suppose that it can function in other signalling pathways. It is noteworthy that trichome formation, which involves some of the same signalling components as root-hair formation, such as GL2 and ZFP5, is influenced by GA signalling. This seems relevant to the discussion, but is not mentioned.

Thank you for pointing this out, we have added a comment on the role of GA in trichome formation in the discussion, line 321 - 328.

The manuscript provides no clues as to the substrate of SPY in epidermal cell patterning and there is clearly more work required.

We agree that it is important to identify direct targets of SPY in this context, but we feel that this is beyond the scope of this manuscript. Very few targets of O-glycosylation have been identified and characterized as yet, as it is technically very challenging. However, we believe that our data nonetheless provide new insights into the regulation of root hair patterning, and strongly point towards an important role for SPY upstream of WER, potentially in cell-to-cell communication between cortex and epidermis. Among potential candidates for direct targets are SCM and JKD, and the latter was already found to be O-GlcNAc modified in a proteomics study (Xu et al. 2017). We did not strongly stress this point in our discussion, as we did not address this experimentally as yet, but future work will certainly go in this direction.

While the conclusions are reasonable, I thought that the evidence for non-involvement of GA signalling in SPY-regulated root hair formation is a little thin. Root development is quite tolerant of changes in GA status. GA levels are close to saturating for root growth, while GA content must be reduced to very low levels before it becomes limiting for root growth. The authors showed no effect of GA application on root hair formation in the wild-type or on the altered cell patterning in the spy-22 mutant. Did they see other changes in root development?

Thank you for your insights. To address this point, we have added Fig. S3 and line 208 - 211, showing overall root length as well as RAM length in Col-0, *spy-22* and *sec-5* grown on different GA concentrations. Both were not significantly affected by the treatment, supporting your argument on GA-levels being close to saturation, and at the same time supporting the hypothesis that patterning defects in *spy-22* might be independent of increased GA-signaling.

In order to suppress GA signalling, they expressed a mutant form of the DELLA protein RGA containing a 17-amino acid deletion, rendering it insensitive to GA-induced degradation. The mutant RGA did not affect root hair formation in Col-0 or spy-22, but what about other aspects of root morphology?

To further characterize root morphology in our *RGA::ΔRGA* lines, we have included Fig. 6 and line 253 - 270, showing that overall root length is decreased in all *RGA::ΔRGA* lines compared to their parents, while RAM length is not statistically significantly affected.

It is unclear whether the increased RGA content in this plant would suppress GA signalling sufficiently. It is also worth considering the potential for the deletion in the mutant RGA altering how it is modified by SPY. It would be worth including severe GA-deficient mutants, which are known to have reduced meristem and final cell size in the root.

Thank you for raising this excellent point. We initially concentrated on the *RGA::ΔRGA* -lines in order to avoid comparing different genetic backgrounds, as our well characterized *spy-22* and *sec-5* lines are in Col-0- background while most available GA-mutants are in Ler. Upon your suggestion, we analysed RAM-length, root length, epidermal cell length and root hair patterning in the GA deficient *ga1-4* and the global *della* mutant (showing constitutively high GA-signaling) compared to wildtype Ler, the data is shown in Fig. S7 and line 274 -287. While RAM and root length were severely affected in *ga1-4*, none of the lines showed any defects in root hair

© 2020. Published by The Company of Biologists under the terms of the Creative Commons Attribution License (https://creativecommons.org/licenses/by/4.0/).

patterning in our hands. We conclude in the discussion that we do not find any evidence that root hair patterning defects in *spy*-22 are GA-dependent, while more experiments are necessary to address a potential interaction of SPY and GA in regulating meristem size (line 338 - 342).

Other minor points:
Line 36: RGA is an abbreviation for REPRESSOR OF ga1-3.

We apologize for this mistake, and have corrected it in line 36 and the abstract.

Line 149: DELLAs can act as transcription co-activators as well as suppressors. Activation of RGA by SPY has the potential to promote gene expression in some physiological contexts.

We have re-phrased the sentence (now line 196 – 198) to:
'So far, the best-characterised target of SPY is the DELLA protein RGA, which undergoes a conformational change upon O-fucosylation that enhances the interaction with downstream transcription factors, *in some cases* inhibiting their binding to DNA (Zentella et al., 2017).'

Lines 173-174: It is not clear whether all growth suppression by DELLAs is due directly to reduced gene expression.

Thank you for this information, we tried to phrase this in a neutral way (now line 243 – 245) by pointing out the effect of DELLAs on growth in general:
'In the current working model, the degradation of DELLAs de-represses DELLA interacting proteins which in turn positively regulate growth (Bao et al., 2020; Davière and Achard, 2016).`

Line 178: The GA receptor is GID1. It should be differentiated from GID2, which, in rice, is the F-box component of the E3 ligase involved in DELLA ubiquitination.

Thank you for pointing this out, we have corrected it in our text.

Second decision letter

MS ID#: DEVELOP/2020/192039

MS TITLE: Arabidopsis O-fucosyltransferase SPINDLY regulates root hair patterning independently of gibberellin signalling

AUTHORS: Krishna Vasant Mutanwad, Isabella Zangl, and Doris Lucyshyn
ARTICLE TYPE: Research Article

I am happy to tell you that your manuscript has been accepted for publication in Development, pending our standard ethics checks.

Reviewer 1

Advance summary and potential significance to field

This has already been indicated in my previous review.

Comments for the author

I am satisfied with authors revisions and have no more to ask for.

© 2020. Published by The Company of Biologists under the terms of the Creative Commons Attribution License (https://creativecommons.org/licenses/by/4.0/).

Reviewer 2

Advance summary and potential significance to field

The authors show that loss of function of the protein O-fucosyltransferase SPINDLY (SPY) results in altered root epidermal cell patterning with ectopic root hair formation. Through examining the effect of the mutation on the expression patterns of transcription factors that determine cell specification, they conclude that SPY acts upstream of GLABRA2 (GL2) and WEREWOLF that regulate formation of non-hair epidermal cells. SPY and the O-linked N-acetylglucosaminetransferase SECRET AGENT (SEC) are known to respectively enhance or suppress the activity of DELLA proteins, which are components of the gibberellin (GA) signalling pathway. The authors show using mutants and hormone application that the action of SPY to regulate root hair formation is independent of GA and ethylene signalling. These are novel findings of general interest in plant developmental biology.

Comments for the author

In this revised manuscript the authors have included further experiments that show convincingly that the action of SPY to regulate root hair cell specification is independent of gibberellin and ethylene signaling.
I have some minor comments on presentation.
Line 41: "the" is unnecessary, unless the authors wish to specificy the GL2 and WER transcription factors.
Line 77:the abbreviation Arabidopsis is used, while later (line 83) it is written in full. In fact, Arabidopsis in roman or italics and in full are used randomly throughout. The abbreviation should be defined on first mention and used consistently.
In Figs 1, 4 and 7, Col-T, Col-A etc, referring to trichoblast and atrichoblast cells should be defined in the figure legends.

© 2020. Published by The Company of Biologists under the terms of the Creative Commons Attribution License (https://creativecommons.org/licenses/by/4.0/).
